# Total Thyroidectomy Versus Lobectomy for Thyroid Cancer: Single-Center Data and Literature Review

**DOI:** 10.1245/s10434-020-09481-8

**Published:** 2021-02-10

**Authors:** Carla Colombo, Simone De Leo, Marta Di Stefano, Matteo Trevisan, Claudia Moneta, Leonardo Vicentini, Laura Fugazzola

**Affiliations:** 1 Division of Endocrine and Metabolic Diseases, IRCCS Istituto Auxologico Italiano, Milan, Italy; 2 Endocrine Surgery Unit, IRCCS Istituto Auxologico Italiano, Milan, Italy; 3 Department of Pathophysiology and Transplantation, University of Milan, Milan, Italy

## Abstract

**Background:**

Controversies remain about the ideal risk-based surgical approach for differentiated thyroid cancer (DTC).

**Methods:**

At a single tertiary care institution, 370 consecutive patients with low- or intermediate-risk DTC were submitted to either lobectomy (LT) or total thyroidectomy (TT) and were followed up.

**Results:**

Event-free survival by Kaplan–Meier curves was significantly higher after TT than after LT for the patients with either low-risk (*P* = 0.004) or intermediate-risk (*P* = 0.032) tumors. At the last follow-up visit, the prevalence of event-free patients was higher in the TT group than in the LT low-risk group (95% and 87.5%, respectively; *P* = 0.067) or intermediate-risk group (89% and 50%; *P* = 0.008). No differences in persistence prevalence were found among microcarcinomas treated by LT or TT (low risk, *P* = 0.938 vs. intermediate-risk, *P* = 0.553). Nevertheless, 15% of the low-risk and 50% of the intermediate-risk microcarcinomas treated by LT were submitted to additional treatments. On the other hand, macrocarcinomas were significantly more persistent if treated with LT than with TT (low-risk, *P* = 0.036 vs. intermediate-risk, *P* = 0.004). Permanent hypoparathyroidism was more frequent after TT (*P* = 0.01). After LT, thyroglobulin (Tg)/thyroid-stimulating hormone (TSH) had shown decreasing trend in 68% of the event-free patients and an increasing trend in the persistent cases.

**Conclusions:**

Lobectomy can be proposed for low-risk microcarcinomas, although in a minority of cases, additional treatments are needed, and a longer follow-up period usually is required to confirm an event-free outcome compared with that for patients treated with TT. On the other hand, to achieve an excellent response, TT should be favored for intermediate-risk micro- and macro-DTCs despite the higher frequency of postsurgical complications.

**Supplementary Information:**

The online version of this article (10.1245/s10434-020-09481-8) contains supplementary material, which is available to authorized users.

An individualized risk-based approach for the treatment of differentiated thyroid cancers (DTCs) focused mainly on a more conservative management has been recommended by guidelines^1^,^2^ in recent years. Controversies remain about the ideal surgical approach because some studies show a higher prevalence of persistent/recurrent disease among patients treated by lobectomy (LT) than among those treated by total thyroidectomy (TT),^3^–^7^ whereas other studies find no significant differences in either disease-free survival (DFS) or overall survival (OS).^8^–^19^ Nevertheless, data in the literature come from a limited number of centers, and very few studies include patients classified according to the American Thyroid Association (ATA) risk stratification system.^10^,^13^,^14^,^17^,^18^ Because follow-up strategies, based primarily on thyroglobulin (Tg) determination, are less reliable for patients treated with LT, TT has been and still is considered the gold standard for the treatment of DTC, and a less aggressive surgical treatment is usually reserved for very-low-risk cases. Consequently, the vast majority of data available on the comparison between the two surgical procedures have been obtained in micro papillary thyroid cancers.^3^,^5^–^9^,^11^,^12^,^15^,^19^ A tentative follow-up approach for patients treated with LT has been proposed that includes a Tg cutoff to indicate persistence or recurrence,^13^ although these data have not yet been confirmed.

The current study aimed to assess possible differences between patients treated with LT or TT and classified as ATA low or intermediate risk. To this purpose, the study evaluated the dynamic risk stratification (DRS), the proportion of patients who needed additional therapies for structural disease, the outcome of the disease, and the prevalence of surgical complications (recurrent laryngeal nerve injury and permanent hypoparathyroidism). The study also aimed to define the predictive role of a Tg trend during the follow-up evaluation of patients treated with LT.

## Materials and Methods

### Study Participants

From 1200 patients followed by a single tertiary care endocrine center during the period 1980–2018, we retrospectively selected 370 consecutive patients.

### Inclusion Criteria

The inclusion criteria specified patients 18 years of age or older at the time of histologic diagnosis who underwent LT or TT as a surgical intervention for DTC.

### Exclusion Criteria

The exclusion criteria ruled out radioiodine residue ablation, surgical treatment after 2018 (follow-up period too short), and ATA high-risk DTC.

### Procedure

Initial thyroid surgery was TT or LT based on the guidelines’ recommendations available at the time of diagnosis. Notably, the indications for performing TT or LT for DTC patients changed several times during the study period,^1^,^2^,^20^–^22^ and LT in particular had a limited indication until the publication in 2016 of the more recent guidelines.

The same surgeon performed the surgery for the majority of the patients. A minority of patients were treated by three different surgeons, all of whom specialized in thyroid surgery, performing surgeries for more than 200 patients per year.

Modified radical therapeutic neck dissection of levels IIa-II-IV, associated with levels IIb and V or not, was performed in cases of clinically apparent abnormal cervical lymphadenopathy, whereas prophylactic neck dissection of the central compartment (level VI) was performed depending on the preference of the individual surgeon. Postoperative laryngeal examination was performed in case of dysphonia, even mild dysphonia, persistent 1 month after surgery. Institutional review board approval was obtained for analysis of the clinical data.

### Risk Stratification

Each patient was risk-stratified according to the seventh edition of the American Joint Commission on Cancer (AJCC)/Union for International Cancer Control (UICC) staging system^23^ and the ATA 2015 Guidelines.^1^

The response to initial therapy (excellent, indeterminate, biochemically incomplete, or structurally incomplete) during the first 2 years of follow-up evaluation was based on the dynamic risk classification (DRS) proposed by Momesso et al.^13^ and reported by other authors.^19^,^24^,^25^

### Follow-up Evaluation

Levothyroxine therapy was routinely prescribed according to TT and LT regardless of post-surgical thyroid function as indicated for moderate iodine-deficient countries.^2^ The levothyroxine dose was titrated to maintain thyroid-stimulating hormone (TSH) levels according to the indications given in different years by authoritative international guidelines,^26^,^27^ and after the publication of the 2015 ATA Guidelines,^1^ according to the ATA risk class and the DRS.

The patients were followed up 1 month after surgery, every 6 months during the first year, and then every 6 to 18 months by a full evaluation including physical examination, measurement of Tg and anti-thyroglobulin autoantibody (TgAb) levels, and neck ultrasound, always performed by the same operator. In particular, recombinant human TSH (rhTSH)-stimulated Tg (exclusively for patients treated with TT) was evaluated from 1997 up to the first months of 2016, when we started to assess patients only by basal ultrasensitive Tg (Elecys Tg II-Roche Diagnostics, Basilea-Switzerland), with an analytical sensitivity of 0.04 μg/L.

### Definitions and Clinical End Points

Because reliable demonstration of remission for patients treated with LT can be difficult due to the lower predictivity of Tg levels, we compared our patients by considering the event-free survival rate, defined as the interval between initial surgery and detection of persistent/recurrent disease.

To identify persistent/recurrent disease, ATA guidelines and Italian consensus recommendations were followed.^1^,^2^ In particular, for the patients treated with TT, the indication was a biochemically incomplete response (negative imaging and detectable basal or stimulated Tg or rising anti-Tg antibody levels) or a structurally incomplete response (evidence of disease at structural or functional imaging at any Tg level, with or without anti-Tg antibodies). For the patients treated with LT, the indication was a biochemically incomplete response (elevated non-stimulated Tg levels not consistent with the presence of a normal thyroid lobe or serum Tg levels, increasing over time in the presence of similar TSH levels, and a negative ultrasonogram [US]) or a structurally incomplete response (structural evidence of disease regardless of serum Tg).

Distant metastases were assessed, by computed tomography (CT), magnetic resonance imaging (MRI), fluorodeoxyglucose positron emission tomography (FDG PET) as appropriate and according to the availability of a given imaging technique at the time of diagnosis, whereas local recurrences were studied by US, cytology, and/or fine-needle aspiration (FNA) Tg measurement.

### Literature Search

We performed a PubMed search for studies published between 2002 and 2019 using the terms “lobectomy,” “hemithyroidectomy,” and “lobo-isthmectomy.” Meanwhile, we checked the references of each included paper and in all available guidelines to identify additional relevant publications.

### Inclusion Criteria for Studies

Studies were included in this series if they compared lobectomy and total thyroidectomy outcomes for patients with differentiated thyroid cancer (i.e., papillary and follicular).

### Exclusion Criteria for Studies

The exclusion criteria ruled out opinions, reviews, commentary, case reports, and studies with insufficient data. Studies also were ruled out if they included thyroid tumors other than differentiated thyroid cancers (i.e., papillary and follicular) or patients submitted to radioiodine remnant ablation. Studies written in languages other than English and those without surgical histopathology results also were excluded.

### Statistical Analysis

Relations between discrete variables were evaluated by means of the Chi square test or *t* test as appropriate. Outcome predictors were evaluated by univariate analysis. Univariate analyses between covariates and risk of recurrence were performed using the Chi square test. The log-rank test was used to examine 20-year Kaplan–Meier curves, with patients censored when an event-free outcome or persistent/relapsing disease was confirmed at the last clinical visit.

Statistical significance was defined as a *P* value lower than 0.05. All statistical analyses were performed using MedCalc Analyses, version 18.11.3 of the MedCalc Software (MedCalc, B-8400 Ostend, Belgium).

## Results

### Clinicopathologic Characteristics of the Cohort and Surgical Complications

We applied ATA 2015 risk stratification to our series to exclude high-risk cases and to classify the remaining cases into low-risk (89%, 331/370) and intermediate-risk (11%, 39/370) DTCs. The prevalence of patients treated with TT was higher in the low-risk group than in the intermediate-risk group (88% vs. 69%; *P* = 0.001). The frequency of post-surgical complications (i.e., recurrent laryngeal nerve injury and permanent hypoparathyroidism) was higher among the patients who underwent TT (8.5%, 27/318) than among those treated with LT (3.8%, 2/52) (*P* = 0.25; Fig. S1, upper panel). In particular, recurrent laryngeal nerve injury was recorded in 23 (7.2%) of the 318 patients who underwent TT and in 2 (3.8%) of the/52 patients treated with LT (*P* = 0.33). Permanent hypoparathyroidism was found in 32 (10%) of the 318 patients who underwent TT and in none of the patients treated with LT (*P* = 0.01; Fig. S1, lower panel). Five patients had both surgical complications. All the patients with paralytic dysphonia underwent logopedical rehabilitation.

*Low*-*Risk Patients* Among the 331 patients in this group, 291 were treated with TT and 40 with LT. The patients treated with LT had a lower median age at diagnosis, had an FTC at histology more frequently, and had a larger mean tumor size (14.5 vs. 9.4 mm; *P* = 0.032) than the patients treated with TT. The percentage of microcarcinomas was 61% (203/331 patients: 182 treated with TT and 21 with LT). Moreover, as expected, multifocality was significantly more prevalent among the patients treated with TT (Table 1). The tumor-node-metastasis (TNM) and AJCC stage did not differ between the two groups. In particular, no metastatic lymph nodes were documented. The mean follow-up period for this group was 79.9 months (median, 59 months; range, 6–475 months).

**Table 1:** Clinicopathologic features of low-risk differentiated thyroid cancers (DTCs) at diagnosis

Low-risk DTCs (*n* = 331)			
Features	DTCs treated with TT	DTCs treated with LT	*P* Value
	(*n* = 291)	(*n* = 40)	
Median age at diagnosis: years (range	49 (18–80)	39 (7–74)	**< 0.0001**
Female/male (%)	234/57 (8/20)	30/10 (75/25)	0.425
Presurgical diagnosis: yes/indeterminate/no (%)	95/35/161 (33/12/55)	14/7/19 (35/17/48)	0.525
Mean size: mm (range)	9.4 (1–40)	14.5 (2–28)	**0.032**
Histology: FTC/PTC (%)	17/274 (6/94)	10/30 (25/75)	**< 0.0001**
Histologic variants of PTC: classical/follicular/other (%)	218/56/0 (80/20/0)	23/7/0 (77/23/0)	0.710
Extrathyroidal invasion: yes/no (%)	0/289 (0/100)	0/40 (0/100)	1
Multifocality: yes/no (%)	84/207 (29/71)	5/35 (12/88)	**0.028**
TNM			
T1/T2/T3/T4 (%)	267/24/0/0 (92/8/0/0)	34/6/0/0 (85/15/0/0)	0.163
N1/N0/NX (%)	0/69/222 (0/24/76)	0/4/36 (0/10/90)	0.142
M1/M0 (%)	0/291 (0/100)	0/40 (0/100)	/
AJCC stage **(1**/2/3/4)	276/12/0/3 (95/4/0/1)	38/2/0/0 (95/5/0/0)	0.788

*TT* total thyroidectomy, *LT* lobectomy, *FTC* follicular thyroid cancer, *PTC* papillary thyroid cancer, *TNM* tumor-node-metastasis, *AJCC* American Joint Committee on Cancer

*Intermediate*-*Risk Patients* This group comprised 39 patients: 27 treated with TT and 12 treated with LT. The patients were highly comparable in terms of baseline clinicopathologic features, without significant differences in age at diagnosis, mean tumor size, histologic features, TNM, or AJCC stage (Table 2). The percentage of microcarcinomas was 41% (16/39), with 11 patients treated by TT and 5 patients treated by LT. Multifocality was recorded in 41% of the TT cases and 17% of the LT cases, although the difference was not statistically significant, likely due to the sample size. Metastatic lymph nodes were found in 15 of the 16 patients treated with TT and lymph node dissection, but these patients were not treated with radioactive iodine (RAI) ablation due to the finding 1 month after surgery of undetectable Tg levels and negative TgAb. The mean follow-up period for this group was 113.1 months (median, 65.5 months; range, 6–483.9 months).

**Table 2:** Clinicopathologic features of intermediate-risk differentiated thyroid cancers (DTCs) at diagnosis

Intermediate-risk DTCs (*n* = 39)			
Features	DTCs treated with TT	DTCs treated with LT	*P* Value
	(*n* = 27)	(*n* = 12)	
Median age at diagnosis: years (range)	47 (15–74)	36.5 (8–70)	0.243
Female/male (%)	23/4 (85/15)	9/3 (75/25)	0.450
Presurgical diagnosis: yes/indeterminate/no (%)	17/2/8 (63/7/30)	7/1/4 (58/8/34)	0.963
Mean size: mm (range)	16.9 (3–47)	17.3 (8–60)	0.910
Histology: FTC/PTC) (%)	2/25 (7/93)	1/11 (8/92)	0.921
Histologic variants of PTC: classical/follicular/other (%)	13/5/7 (52/20/28)	5/3/3 (46/27/27)	0.761
Extrathyroidal invasion: yes/no (%)	11/16 (41/59)	7/5 (58/42)	0.315
Multifocality: yes/no (%)	11/16 (41/59)	2/10 (17/83)	0.146
TNM			
T1/T2/T3/T4 (%)	15/1/11/0 (56/4/40/0)	3/1/7/1 (25/8/59/8)	0.184
N1/N0/NX (%)	15/3/9 (56/11/33)	8/0/4 (67/0/33)	0.470
M1/M0 (%)	0/27 (0/100)	0/12 (0/100)	1
AJCC stage (1/2/3/4)	17/0/7/3 (63/0/26/11)	8/0/3/1 (67/0/25/8)	0.959

*TT* total thyroidectomy, *LT* lobectomy, *FTC* follicular thyroid cancer, *PTC* papillary thyroid cancer, *TNM* tumor-node-metastasis, *AJCC* American Joint Committee on Cancer

### Dynamic Risk Stratification

The response to initial therapy (excellent, indeterminate, biochemically incomplete, or structurally incomplete) during the first 2 years of follow-up evaluation was based on the DRS classification.^13^ The best response during the first 2 years of follow-up evaluation was used to define the response to surgery in both the low- and indeterminate-risk groups (Fig. S2).

*Low*-*Risk Patients* At the 2-year evaluation, 92% (267/291) of the DTCs treated with TT and the 63% (25/40) of those treated with LT had an excellent response (*P* = 0.00001). The prevalence of structurally incomplete responses was higher in the LT group (12%, 5/40) than in the TT group (0.3%, 1/291) (*P* = 0.00001). The prevalence of a biochemically incomplete response was 3% (8/291) in the TT group and 20% (8/40) in the LT group (*P* = 0.00001).

*Intermediate*-*Risk Patients* An excellent response to initial treatment was significantly more frequent among the patients treated with TT (74%, 20/27) than among those treated with LT (33%, 4/12 (*P* = 0.016). No differences were found regarding the prevalence of an indeterminate and biochemically incomplete response, whereas structurally incomplete responses were significantly more frequent among the patients treated with LT (42% [5/12] vs. 7% [2/27]; *P* = 0.010).

### Response to Initial Treatment and Additional Treatments

**Fig. 1:**
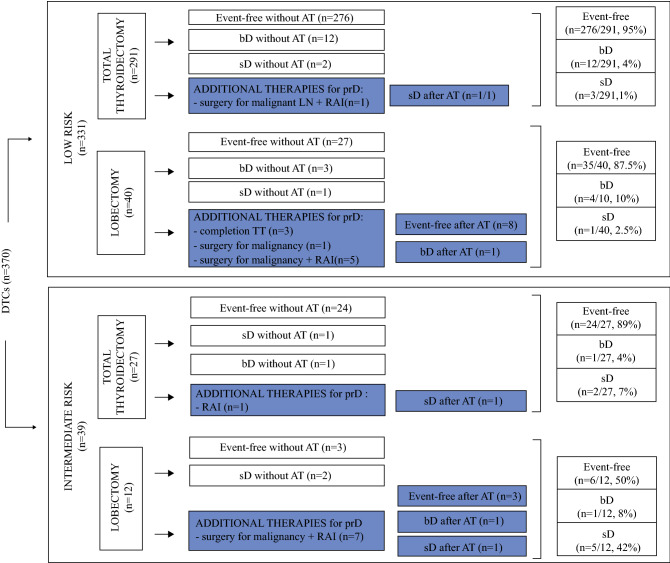
The entire clinical history of the two patient groups, including the first initial treatment and the eventual need for additional treatments up to the report of the final outcome. Additional treatments were performed in case of progressive disease, either biochemical or structural. *AT* additional treatments, *LN* lymph node metastases, *prD* progressive disease, *sD* stable structural disease, *bD* stable biochemical disease

Figure 1 shows the whole clinical history of the two patient groups, including the first surgical treatment and the eventual need for additional treatments up to the confirmed definition of the final outcome (event-free or persistent/relapsing). According to the criteria reported, some patients were considered event-free after surgery, and some had a stable biochemical (bD) or structural (sD) persistence of disease and were thus submitted to an active surveillance, whereas others had progressive disease. These latter patients were given additional treatments (Table S1).

*Low*-*Risk Patients* Among this group, 95% (276/291) of the patients were event-free after TT, whereas 12 patients (4%) had bD, and 3 patients had sD (1%, despite additional treatments in 1 case). On the other hand, the prevalence of event-free patients after LT was 87.5% (35/40), with four patients having bD (10%, despite additional treatments 1 case) and one patient having sD (2.5%).

**Fig. 2:**
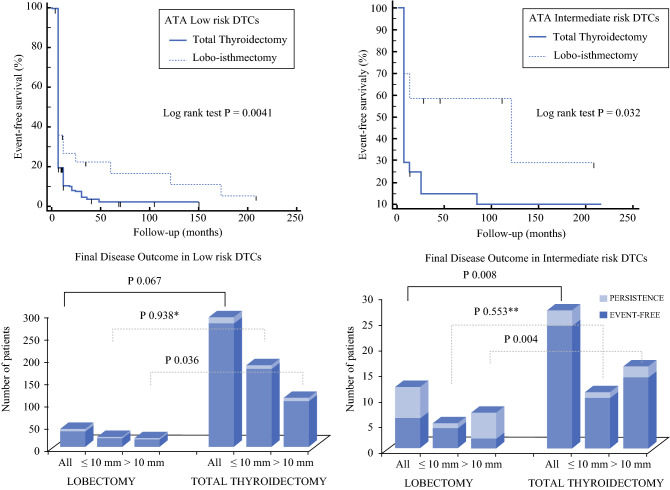
*Upper panel:* 20-year Kaplan–Meier curves by censorship of patients at the time of event-free confirmed definition or in the case of persistent/relapsing disease at the last clinical evaluation. The log-rank test was used to determine the *P* values. The patients who underwent total thyroidectomy had a better remission probability than those who underwent lobectomy (*P* = 0.004 for low-risk differentiated thyroid cancers [DTCs] and *P* = 0.032 for intermediate-risk DTCs). *Among the low-risk microcarcinomas, additional treatments were needed for none of the patients treated with total thyroidectomy (TT) and for 15% of those treated with lobectomy (LT). **Among the intermediate-risk microcarcinomas, additional treatments were needed for none of the patients treated with TT and for 50% of those treated with LT

*Intermediate*-*Risk Patients* Among the patients treated with TT, 89% were event-free at the last follow up visit, whereas 4% (1 patient) had bD and 7% (2 patients) had sD despite additional treatments in 1 case. Among the patients treated with LT, the percentage of event-free cases was lower (50%), with bD in one patient (8%) despite additional treatments, and sD in five patients (42%) despite additional treatments in three cases (Figs. 1 and 2, lower panel). In eight patients treated with LT, suspicious lymph nodes discovered during the surgical procedure were dissected and documented as metastatic at histology. Six of these patients were further treated with completion thyroidectomy plus radioiodine residue ablation, whereas two patients were considered event-free (Fig. 1). Distant metastases were diagnosed during the follow-up period in one patient treated with TT and two patients treated with LT (Table S1).

### Event-Free Survival and Final Disease Outcome

The evaluation of 20-year Kaplan–Meier curves demonstrated that event-free survival was significantly longer for the patients treated with TT than for those treated with LT in both the low-risk ((*P* = 0.004) and intermediate-risk (*P *= 0.032) groups (Fig. 2, upper panel). The outcomes for the two groups of patients at the last follow-up evaluation showed no statistically significant differences between the two surgical procedures for the low-risk patients, although the prevalence of event-free patients was higher in the TT group (95%, 276/291) than in the LT group (87.5%, 35/40) (*P* = 0.067). On the other hand, in the intermediate-risk group, the prevalence of patients with disease persistence/relapse differed significantly between the LT group (50%, 6/12) and the TT group (11% (3/27) (*P* = 0.008) (Fig. 2, lower panel).

Interestingly, after dividing the low- and intermediate-risk cases in both micro- (≤ 1 cm) and macro-carcinoma (> 1 cm), we found no differences in the prevalence of persistence at the final outcome between microcarcinomas treated with LT (5%) or TT (4%) in the low-risk group (*P* = 0.938) and those treated with TT (9%) or LT (20%) in the intermediate-risk group (*P* = 0.553). Nevertheless, all the microcarcinomas treated by TT were event-free after the first surgical treatment, whereas 15% of the low-risk cases and 50% of the intermediate-risk cases treated by LT received additional treatments. On the other hand, macrocarcinomas were significantly more persistent if treated with LT instead of TT (TT [6%] vs. LT [21%] in the low-risk group [*P* = 0.036] and TT [13%] vs. LT [71%] in the intermediate-risk group [*P* = 0.004]) (Fig. 2, lower panel).

### Thyroglobulin/TSH Trend in Patients Treated With Lobectomy

**Fig. 3:**
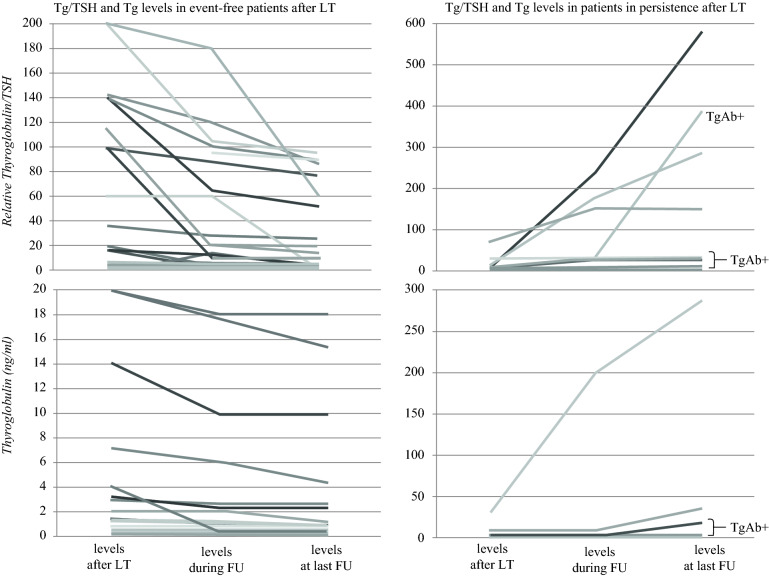
Basal thyroglobulin (Tg) and Tg/thyroid-stimulating hormone (TSH) levels in patients treated with lobectomy (LT) and in remission (*left panel*) or in persistence (*right panel*). The values refer to the evaluation during the follow-up period for the event-free patients (mean, 51 months; median,44 months; range, 12–110 months) and the patients in persistence (mean, 34.5 months; median, 27 months; range, 15–80 months), and at the end of the follow-up period for the event-free patients (mean, 131.4 months; median, 106 months; range, 16–483 months) and the patients in persistence (mean, 135.3 months, median, 110 months; range, 15–324 months). The patients with positive anti-thyroglobulin autoantibodies (TgAb) are shown. Among the 41 event-free patients after LT, a decreasing trend was observed in 28 cases (mean percentage change, − 55% ± 0.27%), whereas among the remaining 13 patients the Tg/TSH ratio remained stable. Four of the patients in persistence showed an increase in the level of Tg/TSH (mean percentage change, + 116% ± 0.95%), whereas among the remaining cases, Tg was stable due to the presence of TgAb

For the patients treated with LT, the levels of serum Tg/TSH immediately after surgery, during the follow-up, and at the last follow-up visit were recorded. In particular, among the 41 event-free patients after LT, we observed a decreasing trend in 28 cases (mean percentage change, –55% ± 0.27), whereas among the remaining 13 patients, the Tg levels and Tg/TSH ratio remained stable (Fig. 3, left panel). Among the patients with bD (*n* = 5) or sD (*n* = 6), we found the levels of Tg and Tg/TSH increased in four cases (mean percentage change 116% ± 0.95), whereas in the remaining cases, Tg was stable due to the presence of TgAb (Fig. 3, right panel, and Table 3).

**Table 3:** Revision of clinical studies published to date on differentiated thyroid cancers (DTCs) treated by total thyroidectomy (TT) or lobo-isthmectomy (LT) without radioactive iodine (RAI)

References	Country	DTCs treated with surgery without RAI				ATA risk (low/intermediate)	Follow-up (months)	Differences in disease outcome
		DTCs treated with TT	DTCs treated with LT	Total DTCs	Hystology			
Appetecchia et al.^8^	Italy	92	28	120	120 micro-PTCs	–	120	No
Pelizzo et al.^3^	Italy	359	44	403	403 micro-PTCs	–	102	Yes (TT better than LT)
Hay et al.^9^	USA	775	125	900	900 micro-PTCs	–	206	No
Vaisman et al.^10^	USA	217	72	289	273 PTCs 10 FTCs 6 Others	214/75	60	No
He et al.^4^	China	225	48	273	273 micro-PTCs	–	162	Yes (TT better than LT)
Ardito et al.^11^	Italy	135	14	149	149 micro-PTCs	–	65	No
Lee et al.^12^	Korea	504	506	1010	1010 micro-PTCs	–	142	No
Pedrazzini et al.^5^	Italy	177	54	231	231 micro-PTCs	–	144	Yes (TT better than LT)
Kim et al.^6^	Korea	1524	3289	4813	4813 micro-PTCs	–	65	Yes (TT better than LT)
Momesso et al.^13^	USA and Brazil	320	187	507	476 PCTs 28 FTCs 3 others	433/74	101	No
Park et al.^14^	Korea	64	293	357	341 PTCs 16 FTCs	187/170	103	No
Dobrinja et al.^15^	Italy	86	19	105	105 micro-PTCs	–	59	No
Kwon et al.^7^	Korea	688	688	1376	1376 micro-PTCs	–	102	Yes (TT better than LT)
Song et al.^16^	Korea	1962	383	2345	2345 PTCs	–	118	No
Liu et al.^17^	China	279	341	620	620 PTCs	0/620	125	No
Geron et al.^18^	Israel	50	109	159	159 PCTs	152/7	103	No
Jeon et al.^19^	Korea	128	127	255	255 microPTCs	–	95	No
Current study (2020)	Italy	318	52	370	340 PTCs 30 FTCs	331/39	84	Yes (TT better than LT for intermediate risk)

*DTC* differentiated thyrid cancer, *PTC* papillary thyroid cancer, *FTC* follicular thyroid cancer, *TT* total thyroidectomy, *LT* lobo-isthmectomy, *ATA* American Thyroid Association, *PTC* papillary thyroid cancer, *FTC* follicular thyroid cancer

### Review of the Literature

We reviewed all the studies published to date, comparing the outcomes between patients treated with LT or TT for low- and intermediate-risk DTCs (Table 3) in a total of 17 studies.^3^–^19^ The publications in the years 2002 to 2019 include three studies from the United States (LT range, 72–187), six studies from Korea (LT range, 127–3289), two studies from China (LT range, 48–341), one study from Israel (LT, 109), five studies from Italy (LT range, 14–54 patients), and no studies from other European countries. The follow-up periods ranged from 59 to 206 months. Interestingly, in 12 of the 17 studies, no differences were found in terms of outcome between the patients treated with LT and those treated with TT, whereas in 5 of the studies, TT was associated with a better outcome. Nevertheless, it should be underscored that only five studies (from the USA, Brazil, Korea, China, and Israel) limited the analysis to low- and intermediate-risk classes.^10^,^13^,^14^,^17^,^18^ Finally, the majority of the studies (11/17) enrolled papillary microcarcinomas exclusively, and follicular thyroid cancers were included in only 3 of the 17 cohorts.

## Discussion

We report data from an Italian series of DTCs divided into ATA low- and intermediate-risk classes and treated with LT or TT. In both classes, after a mean follow up period of 7 years, the patients treated with LT were found to require more additional therapies after initial surgery, to harbor a worse disease response at the dynamic risk stratification, and to have a lower event-free survival according to Kaplan-Meyer curves than the patients treated with TT. In particular, at the last follow-up visit, the prevalence rates for event-free cases after TT and LT were respectively 95% and 87.5% in the low-risk group versus 89% and 50% in the intermediate-risk group. The risk of persistence/relapse for the intermediate-risk patients treated with LT was higher than previously reported,^24^ although scanty and controversial data exist on this topic, mainly because the ATA risk classification is not included in most series.^1^ Discrepancies also could be due to the different proportions of micro- and macrocarcinomas included in the different cohorts. In this context, we found that by dividing our cases into micro- and macrocarcinomas, only the latter were significantly more frequently event-free when treated with TT. However, microcarcinomas did not have a significantly different final outcome. Additional treatments were needed only for the patients treated with LT, especially if belonging to the intermediate-risk category.

Most of the studies available on this topic indicate a lack of differences in outcome between the two surgical procedures, whereas in five large series, including the largest series published to date (4813 patients), TT was associated with a better outcome.^3^–^7^ The differences could be due to several factors including sample size, diagnostic tools (particularly the sensitivity of Tg measurement), the criteria used to define disease persistence, and possibly ethnicity. In this context, the two largest Italian studies found TT associated with a better outcome than LT.^3^,^5^

Surprisingly, no published data on this topic from other European Countries have been published, likely due to the limited indication for LT as a therapeutic option for suspicious cytology or thyroid cancer. Indeed, in Europe, TT is a preferred procedure due to either the higher complexity required for outcome evaluation of patients treated with LT or the relatively huge availability of high-volume endocrine surgeons.

A Tg cutoff of 30 μg/L for patients not treated with levothyroxine after LT has been proposed recently for the definition of persistent or cured disease, but we could not validate this in our series. Indeed, although lower levels are expected in our patients, all receiving thyroxine treatment, the Tg levels were below 10 μg/L in four of six patients with structural disease, indicating that the 30-μg/L threshold would have led to a misdiagnosis in the majority of cases.

On the other hand, the trend of Tg, TgAb, or both was always consistent with US and radiologic findings, allowing correct identification of persistence or confirmation of an event-free outcome for the 40 patients treated with LT. Different data were obtained by Park et al.,^28^ who in a cohort of low-risk patients treated with LT found a progressive increase in Tg/TSH, without differences between cured and recurrent patients, indicating a limited value of Tg trend evaluation for predicting disease recurrence. In contrast to our study, none of the patients in the Korean study was treated with levothyroxine after LT, and a possible effect on Tg reliability can be hypothesized to explain the different results obtained.

The major limitation of the current study was the retrospective nature, as for all the studies published to date on this topic. Although data from a prospective study would certainly be highly significant, our study had the advantage of including only patients followed up in a single tertiary care center, with the result that the clinical record was thus extremely accurate, and information was very rarely missed. Moreover, our study is one of only a few studies and the only study from a European center that included micro- and macro-PTCs as well as a large number of FTCs, all of which had undergone a careful risk stratification and an accurate assessment of the response to therapy in terms of dynamic risk stratification and final disease outcome. Other potential limitations were some statistically significant differences in the clinicopathologic features of the low-risk cases.

Nevertheless, after consideration of these limitations in detail, it can be argued that they had no impact on the final results. In particular, node dissection was more frequently performed for the patients treated with TT, but no differences were found between the two groups at histology because all the removed nodes were negative. Moreover, the tumors treated with LT were larger and more frequently FTC at histology. This could have had an impact on the greater need of additional therapies for the low-risk patients treated with LT versus those treated with TT, but the final outcomes did not differ, supporting the indication for a more conservative treatment in this group of patients, as suggested by the ATA 2015 guidelines,^1^ even in cases with a potentially more aggressive presentation.

Finally, the selection between LT and TT possibly could have been biased by a US presurgical staging. Nevertheless, it appears that this drawback did not occur because the tumors treated with LT were significantly larger (low-risk category) or comparable in size (intermediate-risk category), and no differences were found between the tumors treated with LT and those treated with TT in terms of nodal metastases at histology.

In conclusion, our data indicate that LT can be proposed for low-risk microcarcinomas, although in a minority of cases, additional treatments are needed. Because the evaluation of an event-free outcome for patients treated with LT relies on the evidence of a Tg and/or TgAb trend stably declining, and a negative neck ultrasound, a longer follow-up period is required for a reliable definition of the response to surgery than for patients treated with TT. On the other hand, 50% of the intermediate-risk microcarcinomas treated by LT in this study needed additional treatments after initial surgery. Thus, despite the higher frequency of post-surgical complications, TT should be favored for intermediate-risk tumors.

Due to the high variability of Tg secretion, from either normal or metastatic tissue, even if normalized for TSH levels, a Tg threshold to define outcome in LT cases likely will be difficult to establish. Nevertheless, the Tg trend has been found to correlate strongly with ultrasonographic findings and with the clinical status of the patient, and thus should always be included in the follow-up evaluation of patients treated by either TT without radioiodine residue ablation or by LT.

## Supplementary Information

Below is the link to the electronic supplementary material.

Supplementary material 1 (DOCX 224 kb)
