# Supplementary material for: Total Thyroidectomy Versus Lobectomy for Thyroid Cancer: Single-Center Data and Literature Review
Source: Ann Surg Oncol. 2021 Feb 10;28(8):4334–44. doi: 10.1245/s10434-020-09481-8 (PMC8253713; doi:10.1245/s10434-020-09481-8)

**Supplemental Figure 1:** *upper panel*: prevalence of overall post-operative side effects in patients treated by lobectomy (LT) or total thyroidectomy (TT); *lower panel*: prevalence of recurrent laryngeal nerve injury and permanent hypoparathyroidism in patients treated with LT or TT.

**Supplemental Figure 2:** The response to initial therapy (excellent, indeterminate, biochemical incomplete, or structural incomplete) based on the dynamic risk (DRS) classification^13^ in the low- and intermediate risk classes. The best response during the first two years of follow-up was used to define the response to initial therapy.

**Supplemental Table 1:** Clinicopathological feature of 29 DTCs Initially Treated with Total Thyroidectomy (n = 18) and Lobectomy (n = 11) without

RAI and with Structural or Biochemical Disease Persistence/Recurrence at final follow-up.

| **ID Patient** | **Gender,**  **Age at D** | **Histology,**  **tumor size (mm)** | **TNM,**  **7^th^ AJCC Stage** | **First treatment** | **Additional**  **treatments** | **Final outcome** | **Metastatic site** | **Tg (μg/l) and**  **AbTg (U/l) levels**  **at last FU** | **FU (mo)** |
| --- | --- | --- | --- | --- | --- | --- | --- | --- | --- |
| **Low risk** | | | | | | | | | |
| #2 | F, 65 | FVPTC, 2 | pT1aNXM0 (I) | TT | - | bD | - | 2.47; negative | 70 |
| #3 | M, 67 | FTC, 40 | pT2NXM0 (I) | TT | - | sD | LM | 62; negative | 35 |
| #5 | F, 59 | FVPTC, 16 | pT1bNXM0 (I) | TT | - | bD | - | 0.1; 6 (<4.1) | 30 |
| #6 | F, 34 | CPTC, 12 | pT1bNXM0 (I) | TT | - | bD | - | 0.2; 70 (<60) | 20 |
| #8 | F, 46 | FVPTC, 7 | pT1aNXM0 (I) | TT | - | sD | LM | 9; negative | 28 |
| #9 | F, 37 | FTC, 11 | pT1bNXM0 (I) | TT | - | bD | - | <0.1; 94.2 (<4) | 29 |
| #10 | F, 49 | CPTC, 11 | pT1bN0aM0 (I) | TT+L | - | bD | - | 0.4; 517 (<115) | 68 |
| #11 | F, 55 | FVPTC, 4 | pT1aNXM0 (I) | TT | - | bD | - | 0.4; 121 (<160) | 27 |
| #12 | F, 27 | CPTC, 11 | pT1bNXM0 (I) | TT | - | bD | LM | 0.1; 228 (<60) | 51 |
| #13 | F, 34 | CPTC, 9 | pT1aNXM0 (I) | TT | - | bD | - | 0.2; 25.1 (<4.1) | 41 |
| #14 | M, 78 | FTC, 21 | pT2NXM0 (II) | TT | - | bD | - | 3.38; negative | 60 |
| #15 | M, 31 | CPTC, 14 | pT1bNXM0 (I) | TT | 131I | sD | LM | <0.2; 92.4 (<4.1) | 40 |
| #16 | F, 35 | FVPTC, 35 | pT2NXM0 (I) | TT | - | bD | - | 1; negative | 63 |
| #17 | F, 49 | CPTC, 6 | pT1aNXM0 (I) | TT | - | bD | - | 0; 121 (<115) | 105 |
| #18 | F, 62 | FVPTC, 7 | pT1aNXM0 (I) | TT | - | bD | - | 0; 127 (<115) | 15 |
| #20 | F, 25 | FTC, 30 | pT2N0aM0 (I) | LT+L | cT | bD | - | 0.5; negative | 34 |
| #23 | F, 33 | CPTC, 12 | pT2NXM0 (I) | LT | - | bD | - | 0.1; 477 (<280) | 324 |
| #24 | F, 35 | OPTC, 30 | pT2NXM0 (I) | LT | - | bD | - | <0.1; 263 (<115) | 24 |
| #27 | F, 72 | FTC, 15 | pT1bNXM0 (I) | LT | - | sD | LM | 17.8; negative | 24 |
| #28 | F, 26 | CPTC, 7 | pT1aNXM0 (I) | LT | - | bD | - | <0.1; 206 (<60) | 12 |
| **Intermediate risk** | | | | | | | | | |
| #1 | F, 19 | CPTC, 30 | pT3NXM0 (I) | TT+L | - | bD | - | 0.10; 121 (<60) | 227 |
| #4 | F, 26 | SCPTC, 4 | pT1aNX M0 (I) | TT+L | - | sD | LM | 0.3; negative | 223 |
| #7 | M, 70 | FVPTC, 40 | pT3NXM0 (II) | TT | L + 131I | sD | LM + DM | 100; negative | 25 |
| #19 | F, 8 | OPTC, 15 | pT3N1aM0 (I) | LT | cT + 131I | sD | LM | 0.7; negative | 60 |
| #21 | F, 29 | CPTC, 14 | pT3N1bM0 (I) | LT+L | cT + 131I | sD | DM | 5.6; negative | 110 |
| #22 | F, 31 | FVPTC, 24 | pT3NXM0 (I) | LT | cT + 131I | bD | - | <0.50; 65 (<60) | 27 |
| #25 | F, 41 | FVPTC, 8 | pT3N1aM0 (I) | LT+L | cT + 131I | sD | LM | 0.26; 49 (<30) | 20 |
| #26 | M, 62 | FVPTC, 11 | pT1NXM0 (I) | LT | - | sD | DM | 287; negative | 276 |
| #29 | F, 30 | FVPTC, 25 | pT3NXM0 (I) | LT | - | sD | LM | 34.9; 302 (<115) | 14 |

**Legend:** F Female, M Male, D Diagnosis; PTC Papillary Thyroid Carcinoma; CPTC Conventional PTC, FVPTC Follicular Variant PTC; FTC Follicular Thyroid Carcinoma; SCPTC Sclerosing variant PTC, OPTC oxyphilous PTC, AJCC American Joint Committee on Cancer; TT Total Thyroidectomy, LT Lobectomy, L Lymphadenectomy; cT completion Thyroidectomy; 131I: treatment with radioiodine; bD biochemical persistence of disease; sD structural persistence of disease; Lymph node metastases LM; Distant Metastases DM.


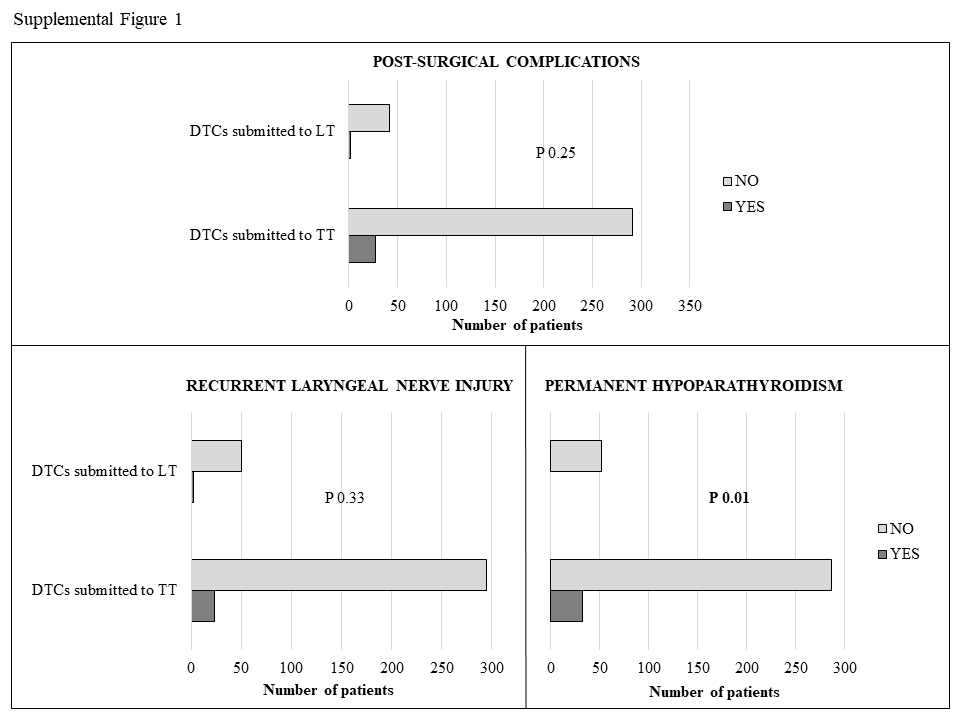


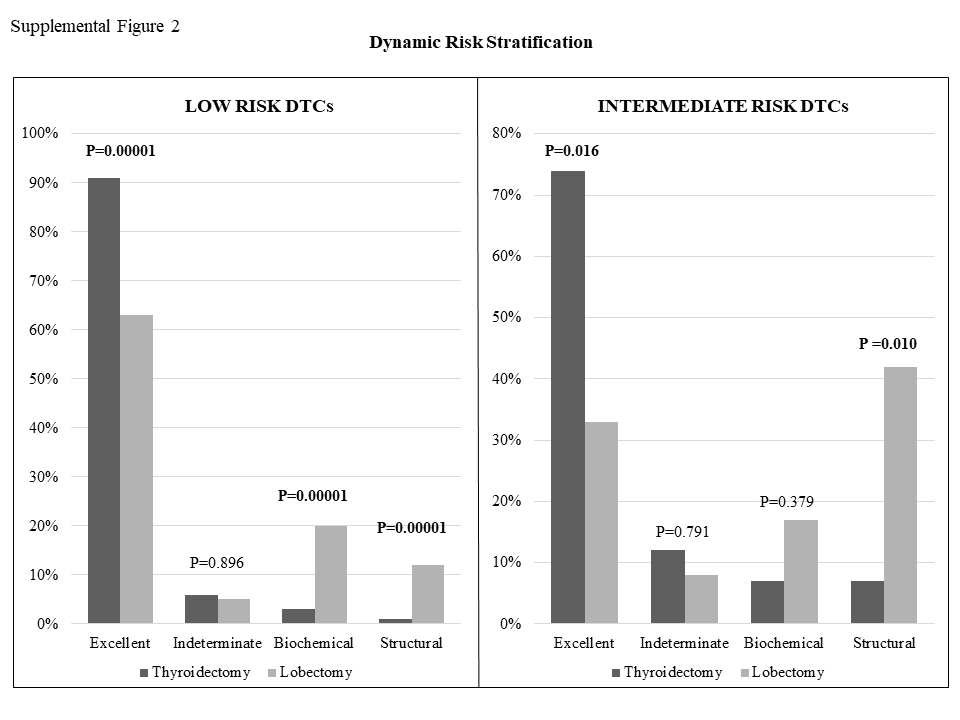

Supplement: Supplementary file 1 — Supplementary material 1 (DOCX 224 kb) [file 10434_2020_9481_MOESM1_ESM.docx]
